# Asciminib resistance of a new BCR::ABL1 p.I293_K294insSSLRD mutant detected in a Ph + ALL patient

**DOI:** 10.1007/s00277-024-06142-8

**Published:** 2025-01-07

**Authors:** Grégoire Cullot, Valérie Lagarde, Jean-Michel Cayuela, Valérie Prouzet-Mauléon, Béatrice Turcq, Yosr Hicheri, Lydia Roy, Thorsten Braun, Marie-Joelle Mozziconacci, Anne-Sophie Alary, Stéphanie Dulucq

**Affiliations:** 1 Univ. Bordeaux, INSERM, BRIC, U1312 Bordeaux, France; 2 Department of Biology, ETH Zurich, Zurich, Switzerland; 3 Laboratory of Hematology, Saint-Louis Hospital, Assistance Publique-Hopitaux de Paris, Université de Paris, University Paris Diderot, Paris, France; 4 CRISP’edit, TBMCore, CNRS UAR3427, INSERM US005, Univ. Bordeaux, Bordeaux, France; 5 Fi-LMC Group, Léon Bérard Center, Lyon, France; 6 Department of Hematology, Institut Paoli-Calmettes, Marseille, France; 7 University Hospital Henri Mondor, AP-HP & Faculté de Santé, UPEC, Service d’Hématologie Clinique, Créteil, France; 8 Department of Hematology Hospital Avicenne, Assistance Publique-Hopitaux de Paris, Bobigny, France; 9 Department of Molecular Biology, Institut Paoli-Calmettes, Marseille, France; 10 Department of Biopathology, Institut Paoli-Calmettes, Marseille, France; 11 Laboratory of Hematology, University Hospital of Bordeaux, Bordeaux, France

**Keywords:** CML, ALL, Cancer resistance, Insertion mutation, Tyrosine kinase inhibitor, BCR::ABL1

## Abstract

Chronic myeloid leukemia and Philadelphia chromosome-positive acute lymphoblastic leukemia patients largely benefit from an expanding tyrosine kinase inhibitors (TKIs) toolbox that has improved the outcome of both diseases. However, TKI success is continuously challenged by mutation-driven acquired resistance and therefore, close monitoring of clonal genetic diversity is necessary to ensure proper clinical management and adequate response to treatment. Here, we report the case of a ponatinib-resistant Philadelphia chromosome-positive acute lymphoblastic leukemia (Ph + ALL) patient harboring a BCR::ABL1 p.I293_K294insSLLRD mutation. Using in vitro proliferation assays on newly generated Ba/F3 cell lines, we confirmed that the mutation confers moderate resistance to ponatinib, and to imatinib and nilotinib. In contrast, *BCR::ABL1*^*SLLRD*^ Ba/F3 cells remain highly sensitive to dasatinib. Unexpectedly, the insertion also provides resistance to asciminib with no inhibitory effect up to 1000 nM. Based on predicted structural models, we speculate that the p.I293_K294insSLLRD disrupts the interaction between the SH3 domain and the kinase domain, shifting the equilibrium toward the active conformation. This shift confers resistance to TKIs that preferentially bind to the inactive conformation, as well as to the allosteric asciminib inhibitor. However, the mutation retains sensitivity to dasatinib, which targets the active form of the kinase.

**Supplementary Information:**

The online version contains supplementary material available at 10.1007/s00277-024-06142-8.

## Introduction

Chronic myeloid leukemia (CML) is a rare hematologic cancer characterized by a massive proliferation of predominately mature myeloid lineage cells [1–4]. CML is defined by the Philadelphia chromosome (Ph +), an acquired t(9;22)(q34;q11) translocation between *BCR* and *ABL1* that produces the constitutively active BCR::ABL1 tyrosine kinase, driving malignant transformation in hematopoietic stem cells [1]. *BCR::ABL1* oncogene is also present in 20–30% of acute lymphoblastic leukemia (Ph + ALL) cases, a rare aggressive lymphoid malignancy that predominantly involves B-lymphocytes [5–9].

The advent of the tyrosine kinase inhibitors (TKIs), with imatinib mesylate as the first, revolutionized CML and Ph + ALL patient treatment by demonstrating outstanding effectiveness [10–16]. However, resistance to imatinib was observed in around 20% of CML patients in chronic phase and for most CML patients in blast crisis or with Ph + ALL [17, 18]. One major mechanism of resistance is the treatment-mediated selection of resistant clones harboring mutations in the BCR::ABL1 kinase domain that impair drug binding [19–22]. Therefore, second-generation (2G) (nilotinib, dasatinib, and bosutinib) and third-generation (3G) (ponatinib) TKIs with more potent binding affinities and different and/or reduced mutational susceptibilities have been developed to overcome this issue.

Nilotinib is derived from imatinib chemical scaffold, but exhibits higher affinity and more potent inhibition of BCR::ABL1 [23]. Both drugs target the ATP-binding site of BCR::ABL1 in its inactive conformation. Dasatinib [23, 24] and bosutinib [25] are dual SRC/ABL1 inhibitor and bind BCR::ABL1 ATP-site in an active conformation. The 3G TKI ponatinib was specifically developed to retain efficacy against the p.T315I mutation while keeping activity against all common BCR::ABL1 mutations in the kinase domain [26–28]. However, ponatinib efficacy is still limited by the occurrence of p.T315I-inclusive compound mutations, or an alternate amino acid substitution at residue p.T315 (p.T315M/L). [28, 29]

The recent development of asciminib opened up a new option for BCR::ABL1 inhibition. Asciminib is an allosteric inhibitor that binds BCR::ABL1 myristoyl pocket, locking the protein in an inactive conformation by mimicking the N-terminal myristoyl moiety [21, 30]. The asciminib binding site is distinct from that of ATP-competitive TKIs, offering two key advantages. First, asciminib has a resistance profile largely non-overlapping with other ATP-competitive TKIs [30, 31]. Second, combining asciminib with an ATP-competitive TKI is likely to overcome most resistance due to point mutations and enhance response, with the asciminib-ponatinib combination demonstrating an unprecedented in vitro response to compound mutations, including the p.T315I mutation. [21, 32]

Nonetheless, two decades of experience with TKIs have shown that mutation-driven resistance is a major escape mechanism that limits treatment success. Therefore, continuous monitoring of the clonal mutational landscape induced by the latest treatments, along with understanding its impact, is crucial for making the best clinical decisions and achieving optimal outcomes.

Here, we report the case of a Ph + ALL patient with a ponatinib-driven selection of a clone harboring a BCR::ABL1 p.I293_K294insSSLRD mutation. In-frame insertion of several amino-acids at this location have been already observed in 4 patients [33–36]. However, their impacts on TKI resistance haven’t been characterized in-depth, especially on the most recently developed BCR::ABL1 inhibitors. In this study, we generated *BCR::ABL1*^*WT*^ or *BCR::ABL1*^*SLLRD*^ Ba/F3 cell lines and performed in vitro proliferation assays to assess their sensitivity to BCR::ABL1 inhibitors. We found that the mutation confers moderate resistance to ponatinib, as observed in the patient, as well as to imatinib and nilotinib. In contrast, *BCR::ABL1*^*SLLRD*^ Ba/F3 cells remain highly sensitive to dasatinib. Unexpectedly, the insertion also conferred resistance to asciminib, showing no inhibitory effect up to 1000 nM.

## Methods

### Cloning

A HIV-1 -based lentiviral vector was engineered to express the p210 isoform of *BCR::ABL1*^*WT*^ and a PGK_tdTOMATO cassette for positive selection of transfected clones (hereafter called p210-*BCR::ABL1*^*WT*^-tdTomato). The p210-*BCR::ABL1*^*SLLRD*^-tdTomato was created by inserting the 5’-AGT CTC CTC AGA GAT-3’ at the p.I293_K294 position (p.I293_K294insSLLRD). For that, a 3.2 kb fragment including p.I293_K294 position was cut from the p210-*BCR::ABL1*^*WT*^-tdTomato using NheI-HF and SgrAI digestion (New England Biolabs). The linear fragment was subcloned into a TOPO BluntEnd vector and modified using In-Fusion cloning (Takara) (**Supplemental Table 1**). Correctly modified clones were selected and digested again with NheI and SgrAI. The 3.2 kb fragment including the 5’-AGT CTC CTC AGA GAT-3’ (p.I293_K294insSLLRD) mutation was then re-inserted in the linearized original vector to create p210-*BCR::ABL1*^*SLLRD*^-tdTomato. The insertion of the 5’-AGT CTC CTC AGA GAT-3’ (p.I293_K294insSLLRD) mutation was verified by Sanger sequencing.

### *BCR::ABL1*^*WT*^ and *BCR::ABL1*^*SLLRD*^ cell line generation

Ba/F3 cell line was purchased from cell repository banks DSMZ (Braunschweig, Germany). Ba/F3 cell line was maintained in RPMI Medium 1640 (Gibco, Paisley, United Kingdom), L-Glutamine (Life Technologies, Grand Island, NY), 25 mM HEPES (Gibco®) supplemented with 10% fetal bovine serum (Eurobio), GlutaMAX (Gibco®), 100 U/mL penicillin, and 100 μg/mL streptomycin. The media was supplemented with 10 ng.mL^−1^ murine IL-3. The Ba/F3 cells were transfected by electroporation using the AMAXA Nucleofector electroporation system (Lonza®, Bale, Switzerland). In brief, 2 million cells were nucleofected with 2 μg of p210-*BCR::ABL1*^*WT*^-tdTomato or p210-*BCR::ABL1*^*SLLRD*^-tdTomato using SG kit and CM147 program. Transfected cells were maintained in media containing 10 ng.mL^−1^ IL-3 for 2 days. Concentration of murine IL-3 was then gradually reduced every two days from 10 to 5 to 2.5 and to 0 ng.mL^−1^ to select for *BCR::ABL1*-positive Ba/F3. Ten days post-IL3 removal, tdTomato-positive cells were sorted by flow cytometry to generate clonal populations. Isolated clones were analyzed by Sanger sequencing to verify *BCR::ABL1*^*WT*^ or *BCR::ABL1*^*SLLRD*^ insertion.

### Ba/F3 proliferation assays

**Fig. 1:**
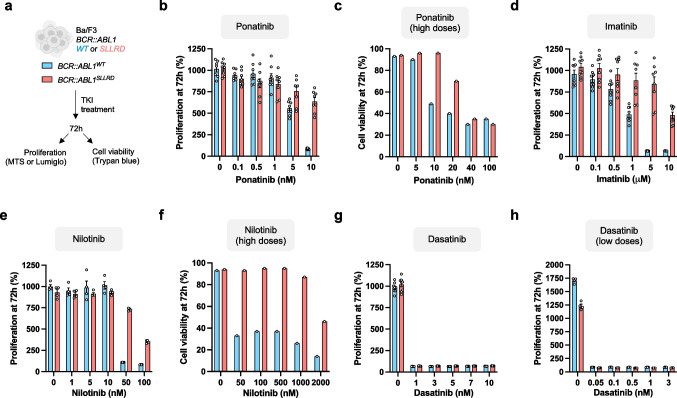
TKI resistance profile induced by the p.I293_K294insSLLRD mutation.** (a)** Experimental workflow for assessing the impact of the p.I293_K294insSLLRD mutation on proliferation or cell viability following TKI treatment. **(b,c)** *BCR::ABL1*^*WT*^ or *BCR::ABL1*^*SLLRD*^ cells treated for 72 h were assessed for proliferation with ponatinib at concentrations ranging from 0 to 10 nM **(b)** and for cell viability at concentrations up to 100 nM **(c)**. **(d)** Proliferation of *BCR::ABL1*^*WT*^ or *BCR::ABL1*^*SLLRD*^ cells after 72-h treatment with imatinib at concentrations up to 10 µM. **(e,f)** Proliferation of *BCR::ABL1*^*WT*^ or *BCR::ABL1*^*SLLRD*^ cells treated for 72 h with nilotinib at concentrations ranging from 0 to 100 nM **(e)** and cell viability assessed at concentrations up to 2000 nM **(f)**. **(g,h)** Proliferation of *BCR::ABL1*^*WT*^ or *BCR::ABL1*^*SLLRD*^ cells treated for 72 h with dasatinib at concentrations ranging from 0 to 10 nM **(g)**, and cell viability assessed at concentrations as low as 0.05 nM **(h)**. Proliferation data are from two independent experiments **(b,d,g)** or one experiment **(e)** with n = 4 replicates for each experiment. Cell viability data are from one independent experiment with n = 1 replicate **(c,f,h)**. Results are presented as mean ± SEM as appropriate

*BCR::ABL1*^*WT*^ and *BCR::ABL1*^*SLLRD*^ Ba/F3 cells were treated for 3 consecutive days with specified TKI type and dose. Proliferation was assessed every day by the MTS (3-(4,5-dimethylthiazol-2-yl)−5-(3-carboxymethoxyphenyl)−2-(4-sulfophenyl)−2H-tetrazolium) tetrazolium assay (CellTiter96 Aq_ueous_, Promega, Madison, WI), which measures viable cells. For that, 3 × 10^3^ cells were plated in quadruplicate into microtiter-plate wells in 100 μL cell culture media with various doses of imatinib, nilotinib, dasatinib, ponatinib and asciminib (Selleckchem, Souffelweyersheim, France). Three hours after adding MTS, the plates were read in a microplate autoreader (Biorad) at 490 nm wavelength to quantify the optical density. The results were normalized to the optical density obtained before exposure to TKIs and expressed as a percentage of proliferation compared to starting condition. All MTS experiments were done in technical quadruplicates. CellTiter-Glo® Luminescent Cell Viability Assay were performed according to manufacturer instructions as a confirmation of MTS experiments **(see **Fig. 1a**)**. Cell viability was measured by acridine orange and propidium iodide staining and quantified using LUNA-FL™ Dual Fluorescence Cell Counter (Logos Biosystems) according to manufacturer instructions.

### Immunoblot detection and quantification

For the immunoblot detection, 5 million Ba/F3 cells were exposed to specified TKI type and dose for 2 h. Protein lysates were then prepared with cell lysis buffer (Cell signaling Technologies) according to manufacturer instructions. Protein concentrations were determined by the Bradford method (DC Protein Assay, Bio-Rad, Hercules, CA). Approximately 25 μg of protein was resolved on 10% sodium dodecyl sulfate–polyacrylamide gel electrophoresis (SDS-PAGE), blotted onto polyvinylidene (PVDF) membranes (Biorad) by semidry electrophoretic transfer, probed with individual primary antibodies (**Supplemental Table 1**), and visualized by the enhanced chemiluminescence (ECL) system (Biorad). Hsp60 was used for normalization.

## Results

### Case report

A 54-year-old man was diagnosed in 2018 with Philadelphia-positive-ALL. He was treated in the Group for Research on Adult Acute Lymphoblastic Leukemia Philadelphia Positive (GRAAPH) protocol in the intensive chemotherapy arm using nilotinib (400 mg *bis in day*) in combination with vincristine/dexamethasone in cycle 1 and 3; aracytine/methotrexate high dose in cycle 2 and 4, followed by 2 interphases with methotrexate and 6 mercaptopurine until stem cell transplantation). He had an undetectable level of minimal residual disease at the end of consolidation, as determined by RT-qPCR. In the absence of familial and HLA-matching donors, he received an autologous hematopoietic stem cell transplantation followed by imatinib maintenance (600 mg/day). Unfortunately, he experienced early relapse at 6 months with two mutations p.E255K and p.T315I identified by Sanger sequencing with a variant allele frequency (VAF) near 20%. A new induction combining hyperfractionated cyclophosphamide, vincristine, adriamycine and dexamethasone (hyperCVAD) and ponatinib was performed. Because of ponatinib toxicity, treatment was stopped, then resumed at reduced dose (initially 45 mg, then 30 mg and 15 mg/day). After achieving complete remission, he underwent an allogeneic stem cell transplantation in October 2019 but a second relapse occurred two months later. Ponatinib was switched to asciminib. A new Sanger sequencing identified a p.I293_K294insSLLRD but not the two previous mutations p.E255K and p.T315I. The patient's condition suddenly worsened and the patient died in February 2020.

### ***BCR::ABL1***^***WT***^ and ***BCR::ABL1***^***SLLRD***^ Ba/F3 cell line generation

To evaluate the effect of p.I293_K294insSSLRD mutation against TKIs, we established two Ba/F3 cell lines carrying either *BCR::ABL1*^*WT*^ or *BCR::ABL1*^*SLLRD*^ mutated variant. We first engineered a plasmid to express the p210 isoform of *BCR::ABL1*^*WT*^ and a PGK_tdTOMATO cassette for positive selection of transfected clones (p210-*BCR::ABL1*^*WT*^-tdTomato). The mutated p210-*BCR::ABL1*^*SLLRD*^-tdTomato version was generated by inserting the 15 nucleotides encoding for the SLLRD mutation by targeted mutagenesis (**see Methods for details**). We next transfected Ba/F3 cells with p210-*BCR::ABL1*^*WT*^-tdTomato and p210-*BCR::ABL1*^*SLLRD*^-tdTomato (**Supplemental **Fig. 1a). Ba/F3 cells are dependent on murine IL-3, but are no longer dependent once *BCR::ABL1* is expressed. We selected BCR::ABL1-positive Ba/F3 cells by removing murine IL-3 from media (see Methods). Ten days post-nucleofection, we sorted dTomato + Ba/F3 to generate clonal populations of *BCR::ABL1*^*WT*^ or *BCR::ABL1*^*SLLRD*^ Ba/F3. Next, we selected three *BCR::ABL1*^*WT*^ and three *BCR::ABL1*^*SLLRD*^ clones. Sequencing analysis of the clones revealed expected insertion of plasmids (**Supplemental **Fig. 1b) and, BCR::ABL1 expression was also confirmed at the protein level (**Supplemental **Fig. 1c). Finally, BCR::ABL1 functionality was evidenced by increased level of tyrosine phosphorylation (**Supplemental **Fig. 1d). For subsequent experiments, *BCR::ABL1*^*WT*^ clone #2 and #3 as well as *BCR::ABL1*^*SLLRD*^ clone #1 and #3 were pooled.

### p.I293_K294insSSLRD mutation provides moderate resistance to 3G TKI ponatinib

As p.I293_K294insSSLRD mutation was observed in ponatinib-treated patient, we first assess the effect of this mutation against this 3G TKI. We treated *BCR::ABL1*^*WT*^ and *BCR::ABL1*^*SLLRD*^ cell lines with 0.5 to 10 nM of ponatinib and quantified proliferation over 3 days using MTS assay (Fig. 1a,b** and Supplemental **Fig. 2a). The p.I293_K294insSSLRD mutation alone did not confer proliferation advantage in absence of ponatinib (133 ± 4.6%, 292 ± 5.8% and 1015 ± 37% at day 1, 2 and 3 for *BCR::ABL1*^*WT*^* versus* 127 ± 4.8%, 312 ± 6.7% and 1020 ± 28% for *BCR::ABL1*^*SLLRD*^). For *BCR::ABL1*^*WT*^, treatment with 5 nM ponatinib negatively impacted proliferation (−46% at 72 h compared to no treatment) while 10 nM totally abolished proliferation (from 100% input to 84 ± 3% at day 3). In contrast, *BCR::ABL1*^*SLLRD*^ cells still actively proliferate in presence of 10 nM ponatinib (637 ± 44% at 72 h), demonstrating increased resistance.

**Fig.2:**
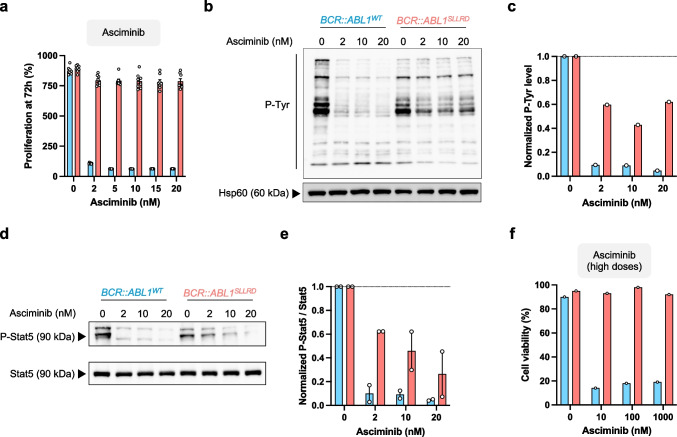
The p.I293_K294insSLLRD mutation induces resistance to asciminib.** (a)** Comparison of proliferation of *BCR::ABL1*^*WT*^ or *BCR::ABL1*^*SLLRD*^ cells treated for 72 h with 0 to 20 nM asciminib. **(b,c)** Detection of phosphorylated tyrosine (P-Tyr) by western blot **(b)** and associated ratio **(c)** in protein lysates extracted after 2 h of asciminib treatment. Hsp60 protein was used as loading control. **(d,e)** Detection of Stat5 and phosphorylated Stat5 by western blot **(d)** and associated ratio **(e)** in protein lysates extracted after 2 h of asciminib treatment. **(f)** Cell viability of *BCR::ABL1*^*WT*^ (left panel) or *BCR::ABL1*^*SLLRD*^ (right panel) cells treated for 72 h with 0 to 1000 nM asciminib. Proliferation data are from two independent experiments with n = 4 replicates for each experiment **(a)**. Cell viability data are from one independent experiment with n = 1 replicate **(f).** Protein level quantification was performed on one **(b)** or two **(d)** western blot replicates from the same lysates. Results are presented as mean ± SEM as appropriate

To explore whether resistance to ponatinib stemmed from a deficiency or decrease in inhibiting BCR::ABL1 kinase activity, we examined the impact of the TKI on Stat5 phosphorylation. For that, *BCR::ABL1*^*WT*^ and *BCR::ABL1*^*SLLRD*^ cells were exposed to increasing doses of ponatinib for 2 h before protein extraction, and we conducted immunoblot analysis of phospho-Stat5 (**Supplemental **Fig. 2b,c). The phosphorylation of Stat5 exhibited a dose-dependent decrease in both *BCR::ABL1*^*WT*^ and *BCR::ABL1*^*SLLRD*^ cells. However, this reduction was less pronounced compared to *BCR::ABL1*^*WT*^, suggesting higher BCR::ABL1 activity in presence of the insertion mutation. Since ponatinib demonstrated efficacy against most mutations at higher concentrations (40 nM) [26], we next assess to which extent p.I293_K294insSSLRD mutation protected *BCR::ABL1*^*SLLRD*^ cells from ponatinib treatment. Again, we observed a reduction in cell viability of *BCR::ABL1*^*WT*^ cells from 10 nM ponatinib (Fig. 1c** and Supplemental **Fig. 2d), with IC50 values of 11.9 nM at 48 h and 8.1 nM at 72 h. In contrast, in the *BCR::ABL1*^*SLLRD*^ cells, the IC50 values increased to 21.7 nM and 22.0 nM at 48 and 72 h, respectively, representing a 1.8-fold and 2.7-fold change. Importantly, higher concentrations (≥ 40 nM) were able to overcome the resistance to ponatinib conferred by the insertion mutation.

Overall, these results confirmed that the p.I293_K294insSSLRD mutation alone is sufficient to induce an increased ponatinib resistance which requires the use of high ponatinib concentrations to block mutated BCR::ABL1 activity.

### p.I293_K294insSSLRD mutation provides resistance to imatinib and nilotinib

In the aforementioned patient’s history, ponatinib treatment was given at relapse because of the emergence of imatinib/nilotinib-resistant p.E255K/T315I clones. However, the p.E255K and p.T315I resistant mutations were not retrieved in ponatinib-resistant p.I293_K294insSLLRD clone suggesting that, the latter might be sensitive to imatinib and nilotinib. In *BCR::ABL1*^*WT*^ cells, decreased proliferation was observed starting from 1 μM imatinib (−46% at 72 h compared to no treatment) and was completely abolished at higher doses (Fig. 1d** and Supplemental **Fig. 3a). In contrast, only reduced proliferation was obtained when treating *BCR::ABL1*^*SLLRD*^ cells with 10 μM imatinib. Similarly, proliferation of *BCR::ABL1*^*WT*^ was suppressed using 50 and 100 nM of nilotinib while significantly detectable in *BCR::ABL1*^*SLLRD*^ (729 ± 7.6% and 315 ± 8.8% for 50 and 100 nM respectively) (Fig. 1e** and Supplemental **Fig. 3b). To confirm resistance of *BCR::ABL1*^*SLLRD*^ cells to nilotinib toward clinically achievable plasmatic concentration of nilotinib [37], we next exposed the cells to up to 2000 nM nilotinib. As previously observed, we confirmed that treatment with nilotinib ≥ 50 nM effectively inhibited the viability of *BCR::ABL1*^*WT*^ cells (Fig. 1f). Conversely, *BCR::ABL1*^*SLLRD*^ cells exhibited profound resistant to nilotinib, being affected only at 2000 nM nilotinib and without reaching the response level of *BCR::ABL1*^*WT*^ cells. Again, these results suggest that p.I293_K294insSSLRD mutation alone is sufficient to induce imatinib and nilotinib resistance.

**Fig.3:**
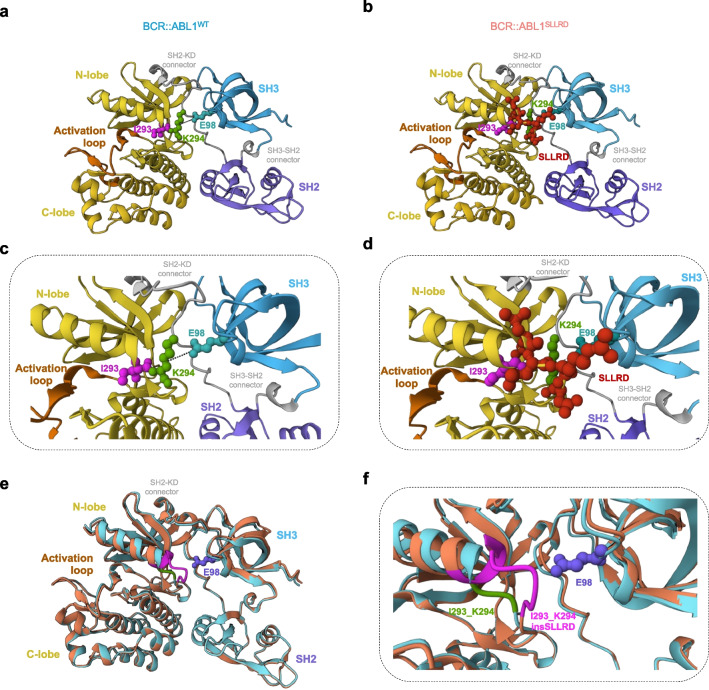
Location of the p.I293_K294insSLLRD mutation**. (a,b)** AlphaFold-predicted ABL1 structure for **(a)** BCR::ABL1^WT^ and **(b)** BCR::ABL1^SLLRD^. **(c,d)** Focus on N-lobe and SH3 interface. In the BCR::ABL1^WT^, p.E98 (located in the SH3 domain) interacts with p.K294 of the N-lobe of the kinase domain (KD). The salt bridge is represented by a black line. In the BCR::ABL1^SLLRD^, the p.I293_K294insSLLRD insertion mutation induced a conformational change of p.K294 (≈ 90° rotation). **(e,f)** Superposition of AlphaFold-predicted ABL1 conformation of BCR::ABL1^WT^ and BCR::ABL1^SLLRD^ **(e)** and focus on the interface between the N-lobe of the kinase domain (KD) and SH3 **(f)**. BCR::ABL1^WT^ and BCR::ABL1^SLLRD^ are represented in cyan and orange respectively. In addition, p.I293_K294, p.I293_K294insSLLRD and p.E98 are highlighted in green, purple and blue respectively. The overall conformation of ABL1 is unchanged by the presence of the p.I293_K294insSLLRD mutation, except at the SH3-KD interface

### p.I293_K294insSSLRD mutation is sensitive to dasatinib

The p.I293_K294insSLLRD mutation is in the SH3 contact region of ABL1 kinase domain. The localization of SLLRD mutation may disfavor the autoinhibited conformation of ABL1 kinase by destabilizing intramolecular interactions. Compared to previous tested TKIs, the dual SRC/ABL1 inhibitor dasatinib binds at the ATP site in an active conformation of the ABL1 kinase domain. We therefore assessed the sensitivity of *BCR::ABL1*^*WT*^ and *BCR::ABL1*^*SLLRD*^ to dasatinib. For both *BCR::ABL1*^*WT*^ and *BCR::ABL1*^*SLLRD*^ cells, treatment with doses from 1 to 10 nM resulted in a complete loss of cell survival (Fig. 1g** and Supplemental Fig. 4a**). Immunoblot analysis of overall tyrosine phosphorylation confirmed decreased BCR::ABL1 activity, even in presence of the p.I293_K294insSLLRD mutation (**Supplemental Fig. 5b,c**). We observed equivalent reduction of phosphorylation of Stat5 with dasatinib treatment in both cell lines (**Supplemental Fig. 5d,e**). Similar cell growth inhibition was obtained using doses down to 0.05 nM dasatinib, a dose that is readily achievable under clinical conditions (Fig. 1h** and Supplemental Fig. 4e**). Altogether, these results demonstrate that the p.I293_K294insSLLRD mutation confers increased resistance to ponatinib, imatinib and nilotinib, but retains sensitivity to dasatinib. The TKI resistance profile conferred by this mutation was confirmed using the CellTiter-Glo luminescent cell viability assay (**Supplemental Fig. 5**).

### p.I293_K294insSSLRD mutation provides resistance to allosteric inhibitor asciminib

We next evaluated the sensitivity of *BCR::ABL1*^*SLLRD*^ Ba/F3 to the allosteric inhibitor asciminib. For *BCR::ABL1*^*WT*^ cells, exposure to 2 nM asciminib is sufficient to completely abolish cell growth (Fig. 2a** and Supplemental Fig. 6a**), whereas *BCR::ABL1*^*SLLRD*^ cell proliferation remains unchanged in the presence of up to 20 nM asciminib. After brief exposure to asciminib, we still observed a substantial level of tyrosine phosphorylation level in *BCR::ABL1*^*SLLRD*^ cells (> 40%), whereas it was largely abolished in *BCR::ABL1*^*WT*^ cells (Fig. 2b,c). We next analyzed phosphorylation level of Stat5. Consistent with proliferation data, Stat5 phosphorylation showed a marked decrease from 2 nM asciminib in *BCR::ABL1*^*WT*^ cells (Fig. 2d,e). In contrast, we observed a dose-dependent reduction of phospho-Stat5 in *BCR::ABL1*^*SLLRD*^ with a maximum 80% reduction in phospho-Stat5 levels after exposure to 20 nM asciminib. We next test if the resistance provided by p.I293_K294insSSLRD could be revert by using higher asciminib doses that can be readily achieve in clinical conditions [21]. Cells were incubated with 10 to 1000 nM asciminib and counted every day to assess viability (Fig. 2f** and Supplemental Fig. 6b**). Strikingly, we observed that *BCR::ABL1*^*SLLRD*^ cells were not affected by asciminib treatment, whereas the viability of *BCR::ABL1*^*WT*^ cells is largely decreased with 10 nM asciminib. Altogether, these results suggests that p.I293_K294insSSLRD mutation also provides resistance to the allosteric inhibitor asciminib.

## Discussion

The advent of TKIs has revolutionized treatment management in CML and Ph + ALL. In parallel, an increasing number of significant genetic variations has been identified and is linked to TKI resistance and failure in a subset of patients. These findings illustrate the importance of characterizing genetic tumor diversity and clonality to ensure the choice of adequate therapy.

Here, we reported the case of a ponatinib-resistant Ph + ALL patient harboring a novel insertion mutation p.I293_K294insSSLRD located in the BCR::ABL1 kinase domain. Four similar insertion mutations (p.I293_K294insAFGS, pK294_H295insRGG, p.K294_H295insH and, p.K294SinsFPQ) have been reported to induce imatinib-resistance while retaining sensitivity to dasatinib [33–36]. However, the effect of these mutations on the latest generation of TKIs has not been investigated. In the present study, we confirmed that p.I293_K294insSSLRD mutation provides significant resistance to imatinib and nilotinib. In vitro proliferation assays confirmed that the p.I293_K294insSSLRD mutation also confers moderate resistance to the 3G TKI ponatinib. Ponatinib resistance conferred by the insertion mutation can be effectively overcome in vitro, using high concentrations (≥ 40 nM) that can be achieve in patients receiving daily doses of ≥ 30 mg. [38] However, administering high doses of ponatinib can potentially result in cardiovascular toxicity [27, 28, 38]. In this case, patients can be managed with lower doses of ponatinib, as in the patient case reported here, which may lead to plasma concentrations where the p.I293_K294insSSLRD mutation is likely to be resistant. In contrast, *BCR::ABL1*^*SLLRD*^ cells remain sensitive to dasatinib, suggesting that these patients may benefit from this TKI. Unfortunately, dasatinib efficacy could not be assessed in the patient described here. Conversely, the p.I293_K294insSSLRD mutation confers a high level of resistance to the allosteric inhibitor asciminib.

The inactive conformation is stabilized through the interaction of the SH2 and SH3 domains with the kinase domain. Based on predicted protein structures, we confirmed that p.I293_K294insSLLRD was located at the interface between SH3 and the kinase domain (Fig. 3a-d). The p.K294 is located in the N-terminal lobe of the kinase domain and forms a salt-bridge to p.E98 from the SH3 domain [22]. Substitution mutations at this position have been reported previously but only confer minor resistance to imatinib (p.K294R) [20] and asciminib (p.K294E) [21, 22]. The proposed model suggests that the insertion of several amino acids at this position further destabilizes the interaction between the SH3 and kinase domains, which is essential for maintaining the kinase in its inactive state, thereby favoring the active conformation.

This is consistent with our data, which show that the *p.I293_K294insSLLRD* mutation confers resistance to ATP-competitive TKIs that bind to the inactive form, while retaining sensitivity to dasatinib, which preferentially binds to the ATP site in the active conformation. Additionally, the mutation is located far from the asciminib binding site, making it unlikely to directly impair asciminib binding. However, disruption of the interaction between the SH3 domain and the N-lobe of the kinase domain is likely to confer resistance by affecting the allosteric effect induced by asciminib. A similar mechanism has been reported for the *p.M244V* mutation, which impairs the allosteric effect of asciminib by stabilizing the interaction between the SH2 domain and the N-lobe of the kinase domain. [39]

The combined use of an allosteric and an ATP-competitive TKI has shown promising results against compound mutations [21, 32, 40]. However, this approach may not be directly translatable to cases involving resistance-conferring N-lobe mutations.

While Ph + ALL patients typically express the p190 isoform, here we used p210 isoform to align with the approach of most in vitro sensitivity profile studies. [29, 32, 41–43]

Interestingly, imatinib-resistant insertion mutation at this position has been observed in a Ph + ALL patient (as reported here), but also in a patient with biphenotypic acute leukemia [33] and in CML cases [33, 34]. Retrospective analysis of patients managed by the co-authors of this study has brought two additional cases of insertion mutations at the same position.

A patient who received low-dose chemotherapy and ponatinib encountered a cytologic relapse at month 14, characterized by a p.I293_K294insNAPEA insertion mutation. Subsequently, the individual underwent treatment with dasatinib at a daily dose of 100 mg, combined with mini HyperCVAD only 3 weeks after dasatinib treatment start due to the onset of heart failure. Unfortunately, the patient died from blastic aplasia one week later, marking four weeks from the start of dasatinib therapy.

The second patient showed a different pattern. After receiving dasatinib treatment, this patient presented with a p.I293_K294insTG insertion mutation. Notably, subsequent administration of ponatinib resulted in the disappearance of the insertion mutation and the expansion of a compound clone featuring the p.Y253H/p.T315I mutations.

Collectively, these observations highlight the significance of the p.I293_K294 hotspot for resistant insertion mutations. However, discrepancies in TKIs resistance profile observed in the two last Ph + ALL patients could arise from variations in insertion size (only 2 amino acid insertion for the p.I293_K294insTG) and/or ALL-related mechanisms. We examined the resistance and sensitivity profile of the p.I293_K294SLLRD insertion mutation within a CML context. However, inhibition of BCR::ABL1 tyrosine kinase in Ph + ALL is insufficient to suppress leukemic cell growth and require more comprehensive and potent therapeutic approaches to achieve the eradication of Ph + ALL.^44^ This could explained the limited response observed in the patient with the p.I293_K294insNAPEA mutation, predominantly treated with dasatinib alone. Furthermore, despite a favorable initial response of the majority of patients with first-relapsed Ph + ALL to salvage therapy in combination with TKIs, their overall prognosis remains unfavorable. As a result, it becomes challenging to regard these clinical cases as contradictory to our in vitro findings. The latter likely remain valuable and informative, especially in the context of the latest BCR::ABL1 inhibitors that have been developed recently.

In conclusion, this study emphasizes the necessity for a systematic exploration of mutations not only limited to substitutions, but also encompassing insertions and deletions within the tyrosine kinase domain of ABL1.

## Supplementary Information

Below is the link to the electronic supplementary material.

Supplementary file1 (PDF 2396 KB)

Supplementary file2 (PDF 2189 KB)

## Data Availability

Data is provided within the manuscript or supplementary information files. For original data, please contact gregoire.cullot@biol.ethz.ch and/or stephanie.dulucq@chu-bordeaux.fr.
