# Supplementary material for: Asciminib resistance of a new BCR::ABL1 p.I293_K294insSSLRD mutant detected in a Ph + ALL patient
Source: Ann Hematol. 2025 Jan 7;104(2):1117–26. doi: 10.1007/s00277-024-06142-8 (PMC11971149; doi:10.1007/s00277-024-06142-8)
Supplement: Supplementary file 1 — Supplementary file1 (PDF 2396 KB) [file 277_2024_6142_MOESM1_ESM.pdf]

Supplementary Materials

Asciminib resistance of a new BCR::ABL1 p.I293_K294insSSLRD mutant detected in a Ph+ ALL patient.

Grégoire Cullot[1,2,*], Valérie Lagarde[1], Jean-Michel Cayuela[3,5], Valérie Prouzet-Mauléon[1,4], Béatrice Turcq[1,4,5], Yosr Hicheri[6], Lydia Roy[5,7] , Thorsten Braun[8], Marie-Joelle Mozziconacci[9], Anne-Sophie Alary[10#], Stéphanie Dulucq[1,5, 11,*,#]

1 - Univ. Bordeaux, INSERM, BRIC, U1312, Bordeaux, France

2 - Department of Biology, ETH Zurich, Zurich, Switzerland

3 - Laboratory of Hematology, Saint-Louis Hospital, Assistance Publique-Hopitaux de Paris, Université de Paris, University Paris Diderot, Paris, France

4 - CRISP'edit, TBMCore, CNRS UAR3427, INSERM US005, Univ. Bordeaux, Bordeaux, France

5 - Fi-LMC group, Léon Bérard center, Lyon, France

6 - Department of Hematology, Institut Paoli-Calmettes, Marseille, France

7- University Hospital Henri Mondor, AP-HP & Faculté de Santé, UPEC, Service d'Hématologie Clinique, Créteil, France

8- Department of Hematology Hospital Avicenne, Assistance Publique-Hopitaux de Paris, Bobigny, France

9 - Department of Molecular Biology, Institut Paoli-Calmettes, Marseille, France

10-. Department of Biopathology, Institut Paoli-Calmettes, Marseille, France

11-, Laboratory of Hematology, University Hospital of Bordeaux, Bordeaux, France

# - Equal contribution

** - Co-corresponding authors (gregoire.cullot@biol.ethz.ch and stephanie.dulucq@chu-bordeaux.fr)*

Supplemental Figure 1

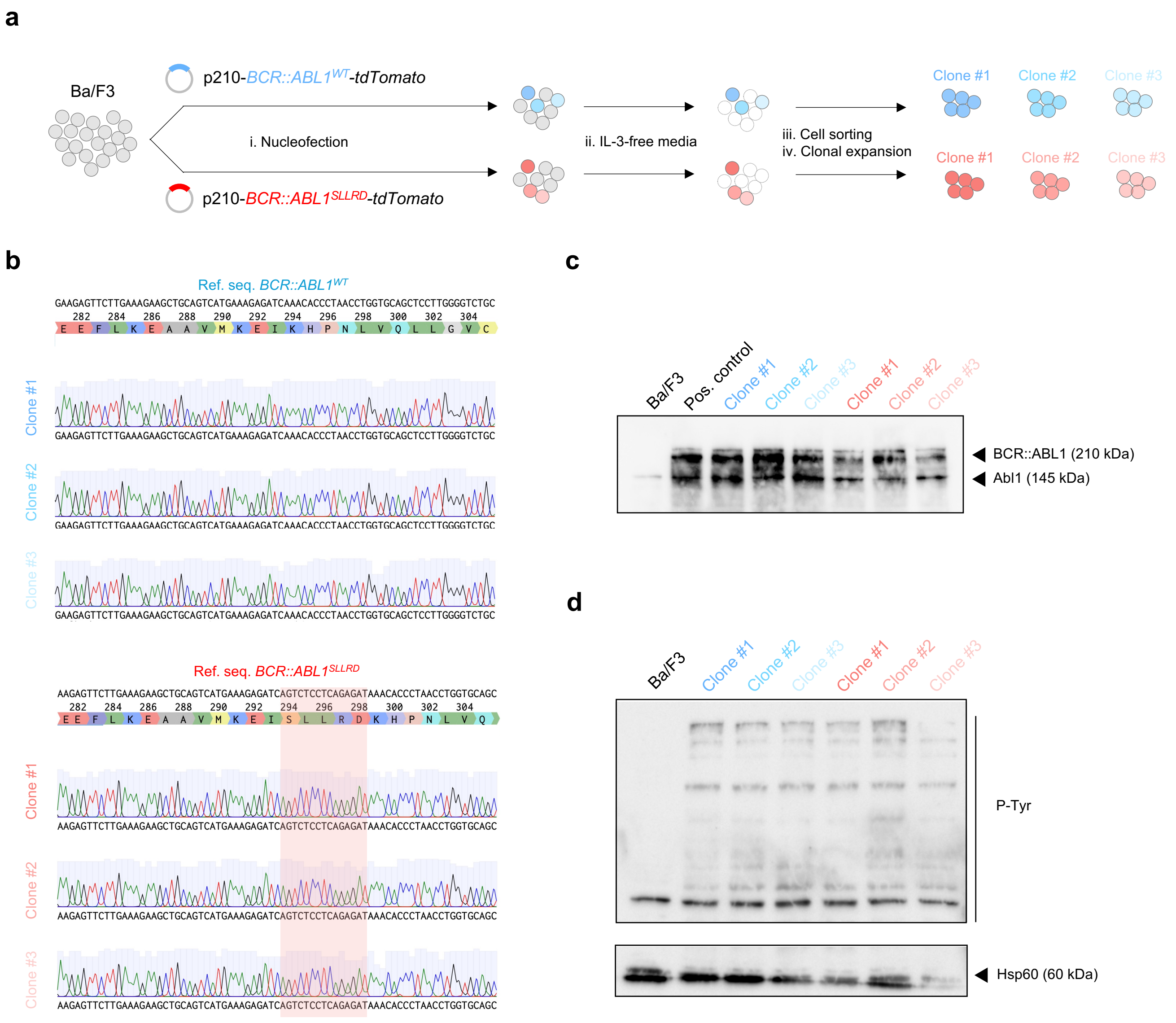

Supplemental Figure 1 - Generation of *BCR::ABL1*^{WT} and *BCR::ABL1*^{SLLRD} Ba/F3 cell lines. **(a)** Experimental workflow for Ba/F3 transfection, *BCR::ABL1*-positive selection, and generation of clonal population by FACS. **(b)** Sanger sequencing of three *BCR::ABL1*^{WT} clones (top, in blue) and three *BCR::ABL1*^{SLLRD} clones (bottom, in red). The insertion of the 15 nucleotides encoding for the p.I293_K294insSLLRD mutation is highlighted in red. **(c,d)** Detection of BCR::ABL1 **(c)** and phosphorylated tyrosine (P-Tyr) **(d)** by western blot. The anti-Abl1 antibody recognized both murine Abl1, endogenously expressed, and human BCR::ABL1. Positive control was made of a lysate from the bulk Ba/F3 nucleofected with p210-BCR::ABL1^{WT}-*tdTomato*.

Supplemental Figure 2

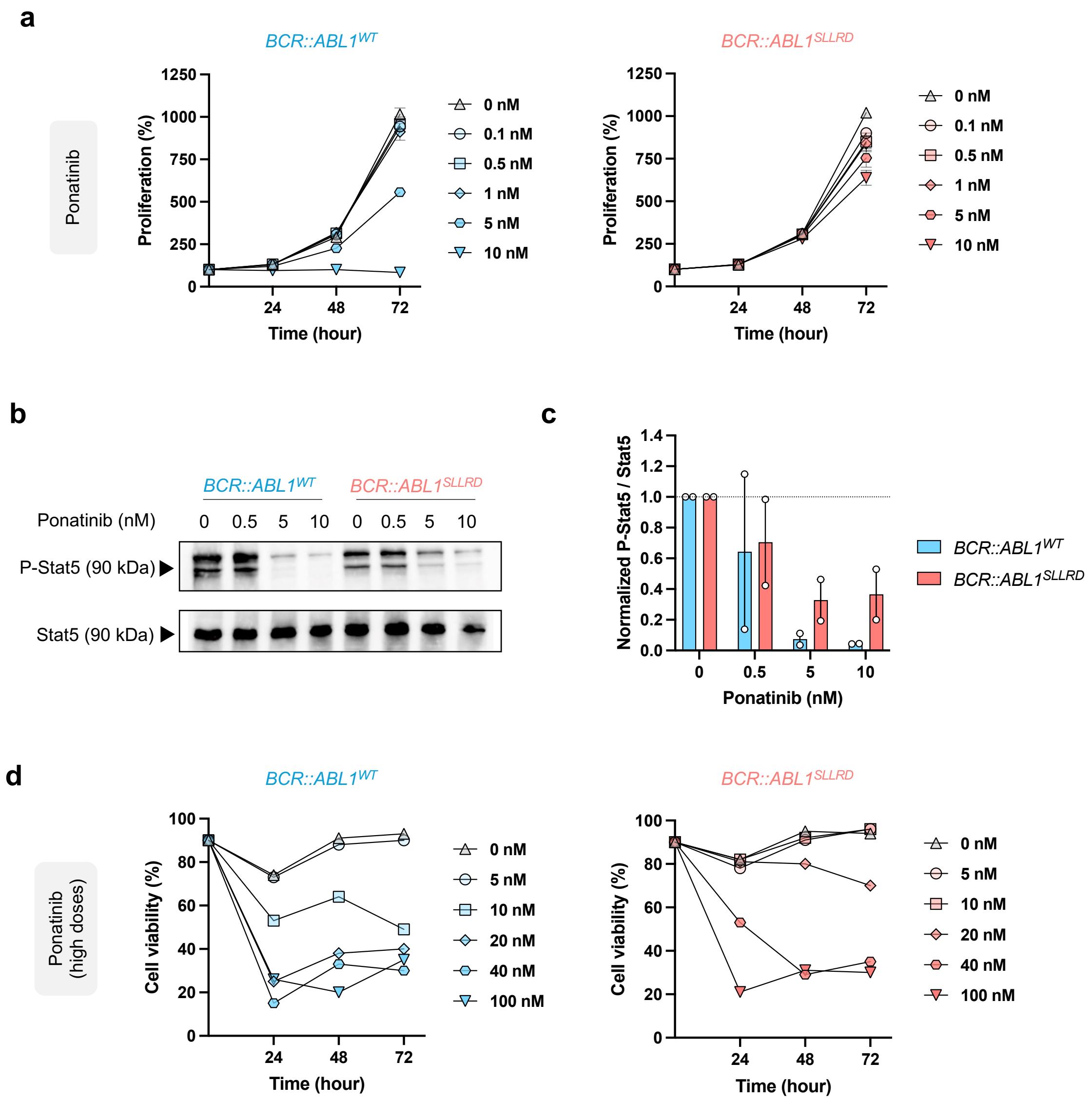

Supplemental Figure 2 - The p.I293_K294insSLLRD mutation induces moderate ponatinib resistance. (a) Proliferation of *BCR::ABL1*^{WT} (left panel) or *BCR::ABL1*^{SLLRD} (right panel) cells treated for 72 hours with 0 to 10 nM ponatinib. **(b,c)** Detection of Stat5 and phosphorylated Stat5 (P-Stat5) by western blot **(b)** and associated ratio **(c)** in protein lysates extracted after 2 hours of ponatinib treatment. **(d)** Cell viability of *BCR::ABL1*^{WT} (left panel) or *BCR::ABL1*^{SLLRD} (right panel) cells treated for 72 hours with 0 to 100 nM ponatinib. Proliferation data are from two independent experiments with n=4 replicates for each experiment **(a)**. Cell viability data are from one independent experiment with n=1 replicate **(d).** Protein level quantification was performed on two western blot replicates from the same lysates **(c)** . Results are presented as mean ± SEM as appropriate.

Supplemental Figure 3

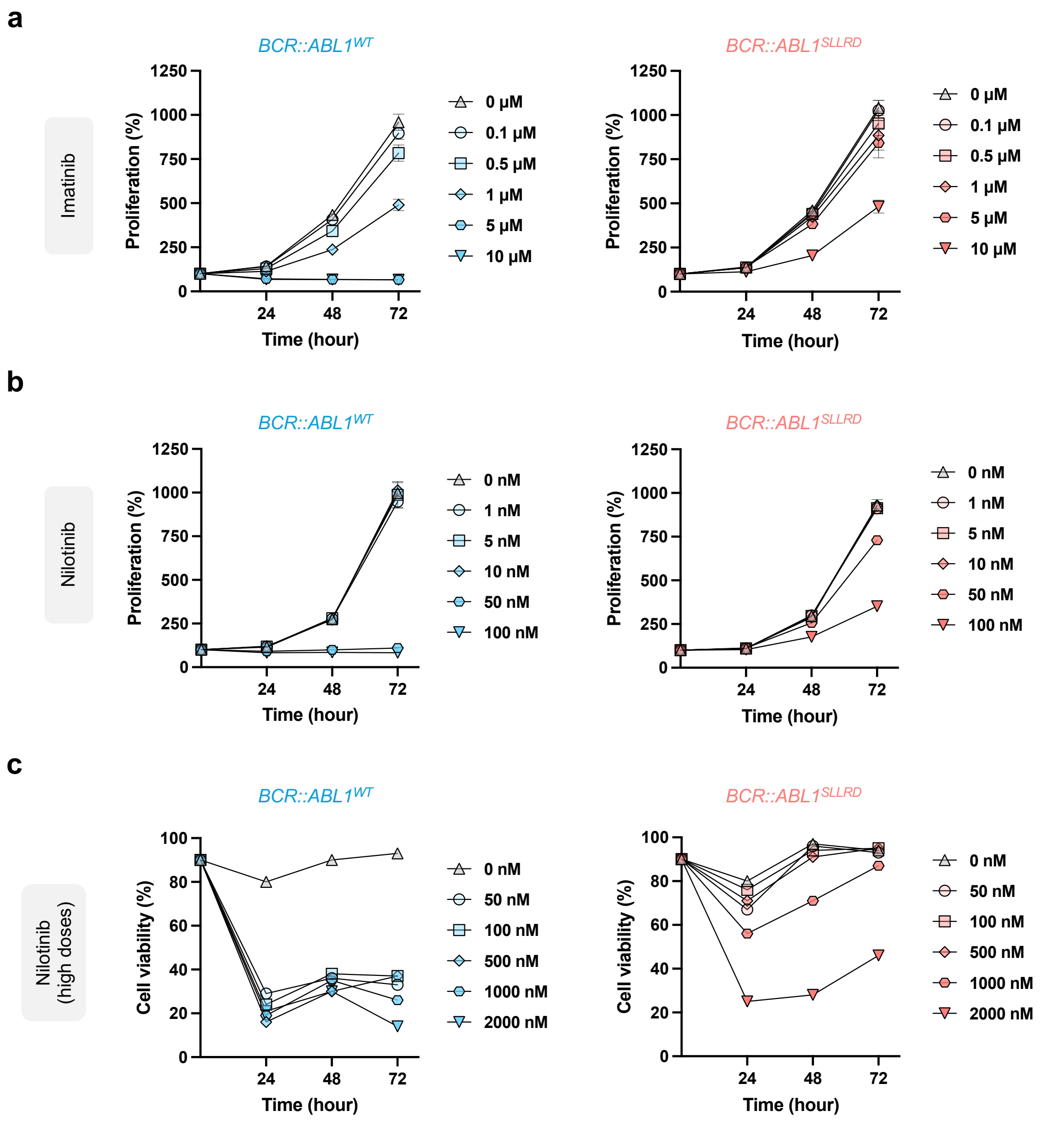

Supplemental Figure 3 - The p.I293_K294insSLLRD mutation induces resistance to imatinib and nilotinib. (a,b) Proliferation of *BCR::ABL1*^{WT} (left panel) or *BCR::ABL1*^{SLLRD} (right panel) cells treated for 72 hours with 0 to 10 µM imatinib **(a)** or with 0 to 100 nM nilotinib **(b)**. **(c)** Cell viability of *BCR::ABL1*^{WT} (left panel) or *BCR::ABL1*^{SLLRD} (right panel) cells treated for 72 hours with 0 to 2000 nM nilotinib. Proliferation data are from two **(a)** or one **(b)** independent experiments with n=4 replicates for each experiment. Cell viability data are from one independent experiment with n=1 replicate **(c).** Results are presented as mean ± SEM as appropriate.

Supplemental Figure 4

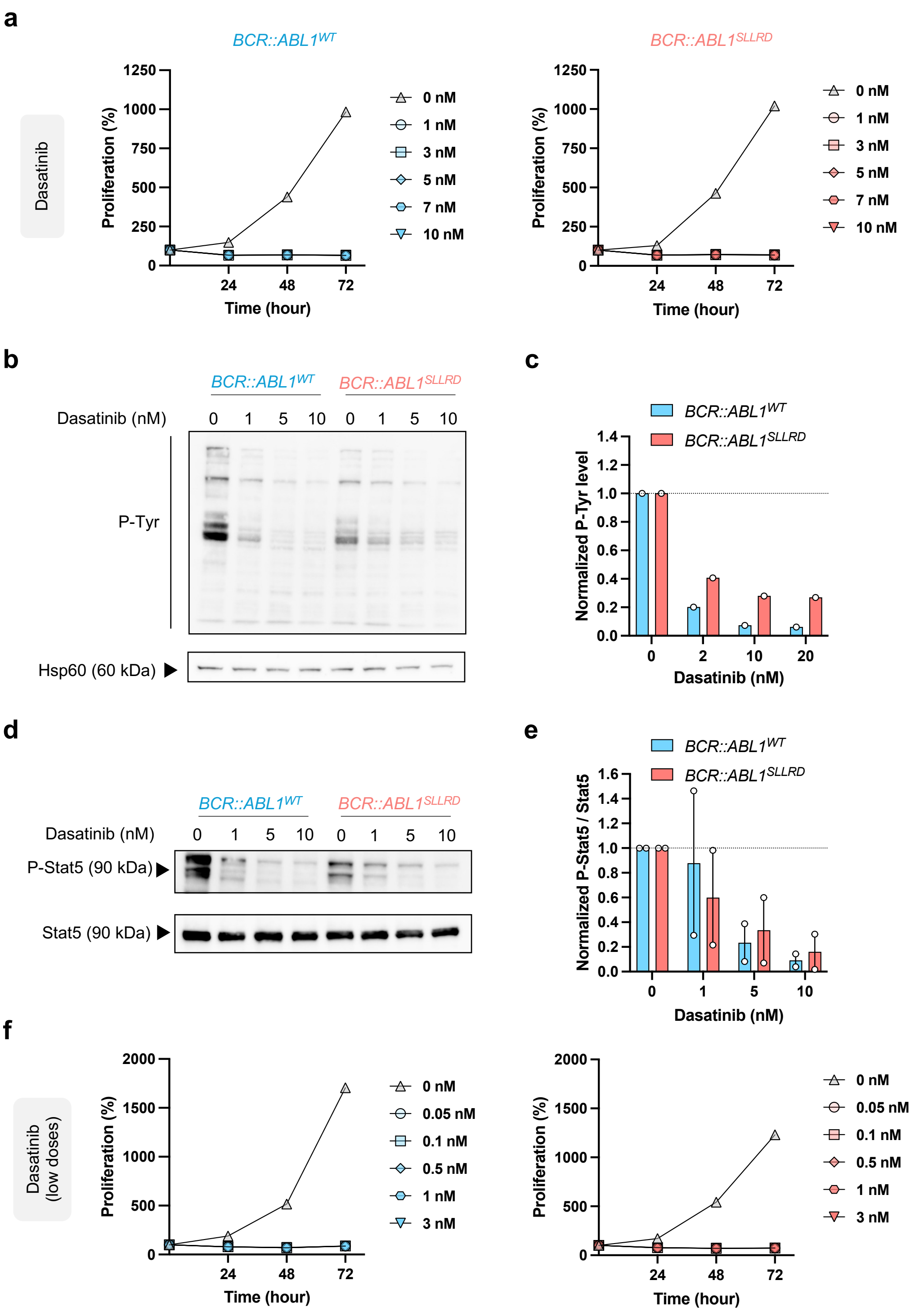

Supplemental Figure 4 – The p.I293_K294insSLLRD mutation is sensitive to dasatinib. (a) Proliferation of *BCR::ABL1^{WT}* (left panel) or *BCR::ABL1^{SLLRD}* (right panel) cells treated for 72 hours with 0 to 10 nM dasatinib. **(b,c)** Detection of phosphorylated tyrosine (P-Tyr) by western blot **(b)** and associated ratio **(c).** Hsp60 protein was used as loading control. **(d,e)** Detection of Stat5 and phosphorylated Stat5 by western blot **(d)** and associated ratio **(e)** in protein lysates extracted after 2 hours of dasatinib treatment. **(f)** Cell viability of *BCR::ABL1^{WT}* (left panel) or *BCR::ABL1^{SLLRD}* (right panel) cells treated for 72 hours with 0 to 3 nM dasatinib. Proliferation data are from two independent experiments with n=4 replicates for each experiment **(a)**. Cell viability data are from one independent experiment with n=1 replicate **(f).** Protein level quantification was performed on one **(c)** or two **(e)** western blot replicates from the same lysates. Results are presented as mean ± SEM as appropriate.

Supplemental Figure 5

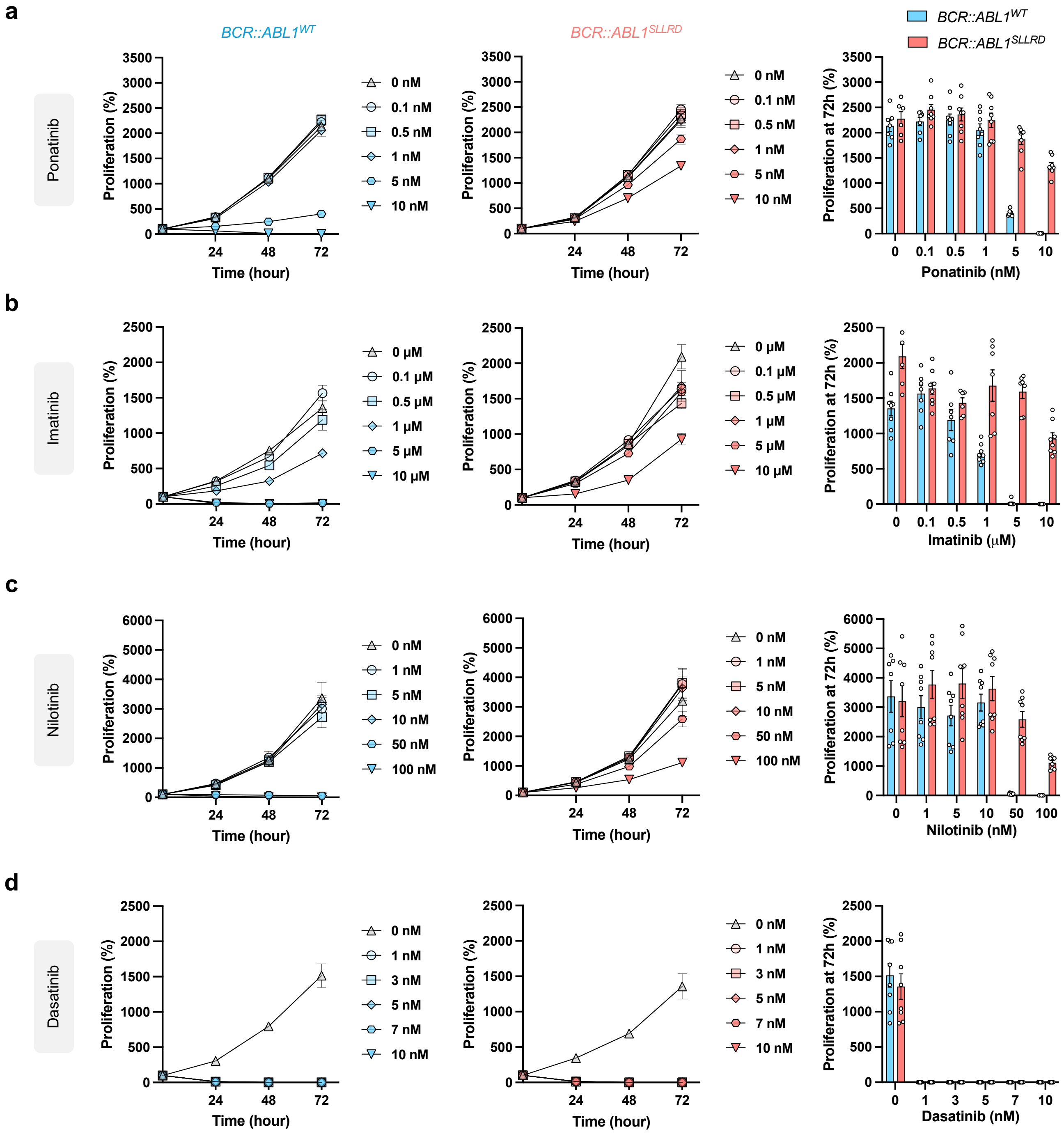

Supplemental Figure 5 – Effect of the p.I293_K294insSLLRD mutation against tyrosine kinase inhibitors. (a-d) Proliferation of *BCR::ABL1*^{WT} (left panel) or *BCR::ABL1*^{SLLRD} (middle panel) cells treated for 72 hours with **(a)** ponatinib, **(b)** imatinib, **(c)** nilotinib and **(d)** dasatinib quantified using CellTiter-Glo® Luminescent Cell Viability Assay. (Right panel) comparison of proliferation results at 72 hours between *BCR::ABL1*^{WT} and *BCR::ABL1*^{SLLRD} cells. Proliferation data are from two independent experiments performed with n=4 replicates for each experiment **(a-d)**. Results are presented as mean ± SEM as appropriate.

Supplemental Figure 6

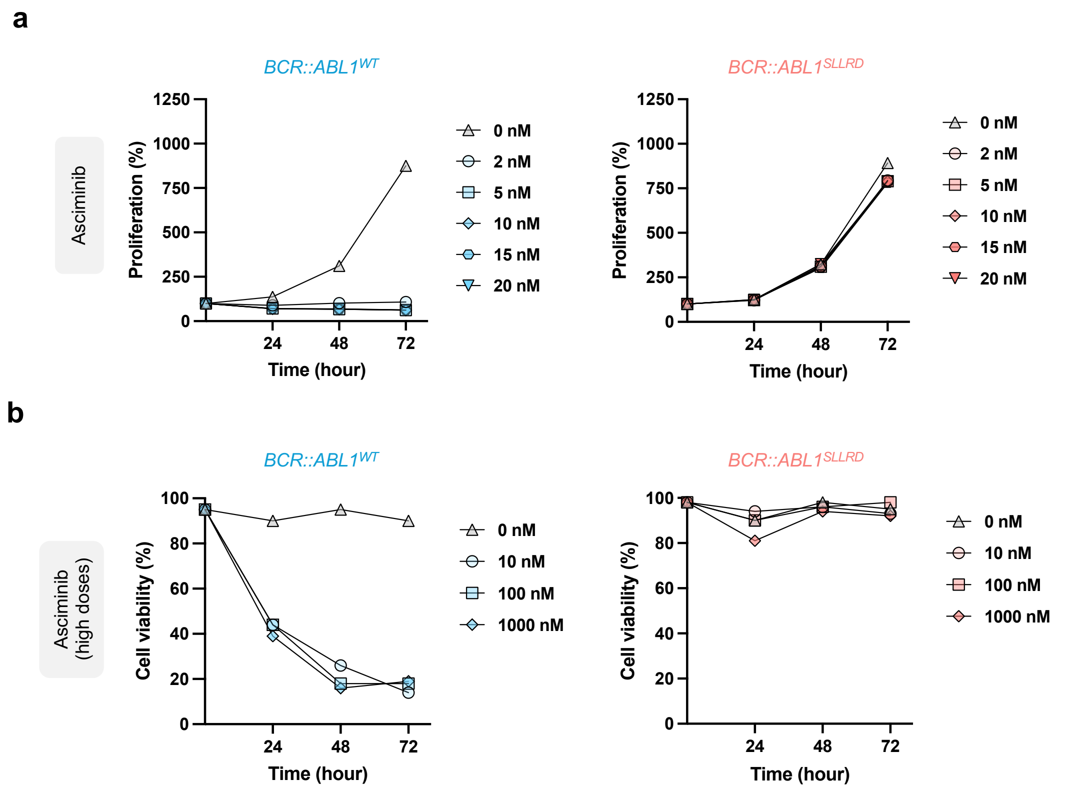

Supplemental Figure 6 - The p.I293_K294insSLLRD mutation induces resistance to asciminib. (a) Proliferation of *BCR::ABL1*^{WT} (left panel) or *BCR::ABL1*^{SLLRD} (right panel) cells treated for 72 hours with 0 to 20 nM asciminib. **(b)** Cell viability of *BCR::ABL1*^{WT} (left panel) or *BCR::ABL1*^{SLLRD} (right panel) cells treated for 72 hours with 0 to 1000 nM asciminib. Proliferation data are from two independent experiments performed with n=4 replicates for each experiment **(a)**. Cell viability data are from one independent experiment with n=1 replicate **(b).** Results are presented as mean ± SEM as appropriate.

Supplemental Table 1

REAGENTS or RESOURCE	SOURCE	IDENTIFIER or SEQUENCE
**Primary antibodies**		
Anti-phosphotyrosine (P-Tyr) (4G10)	Millipore	05-321
Anti-phospho-Stat5 (Tyr694)	Cell Signaling	#9351
Anti-Stat5 (C-17)-G	Santa Cruz Biotechnology	sc-835-G
Anti-phospho-Crkl (Tyr207)	Cell Signaling	#3181
Anti-Hsp60 (K-19)	Santa Cruz Biotechnology	sc-1722
Anti-Abl (8E9)	BD Pharmigen	554148
**Secondary antibodies**		
Rabbit Anti-Mouse Immunoglobulins/HRP	Agilent Dako	P0260
Swine Anti-Rabbit Immunoglobulins/HRP	Agilent Dako	P0217
**Cell line**		
Ba/F3	DSMZ	ACC 300
**Tyrosine kinase inhibitors**		
Imatinib	Novartis	-
Nilotinib	Selleckchem	-
Ponatinib	Selleckchem	-
Dasatinib	Selleckchem	-
Asciminib	Novartis	-
**Critical Commercial Assays**		
CellTiter96 Aq_{ueous} One Solution Cell Proliferation Assay (MTS)	Promega	G3582
CellTiter-Glo Luminescent Cell Viability Assay	Promega	G7570
**Oligonucleotides (5' - 3')**		
In-Fusion BCR::ABL1-SLLRD forward primer	Euromedex	cagagatAAACACCCTAACCTGGTG
In-Fusion BCR::ABL1-SLLRD reverse primer	Euromedex	aggagactGATCTCTTTCATGACTGCAG
Sanger I293_K294 forward primer	Euromedex	TGACCAACTCGTGTGTGAAACTC
Sanger I293_K294 reverse primer	Euromedex	CAGTATCTCAGACGAAGTGGAAAA
**Molecular Cloning**		
In-Fusion seamless cloning	Takara	638948
NheI-HF	NEB	R3131S
SgrAI	NEB	R0603S
