# Supplementary material for: Asciminib resistance of a new BCR::ABL1 p.I293_K294insSSLRD mutant detected in a Ph + ALL patient
Source: Ann Hematol. 2025 Jan 7;104(2):1117–26. doi: 10.1007/s00277-024-06142-8 (PMC11971149; doi:10.1007/s00277-024-06142-8)

Supplementary Materials

Asciminib resistance of a new BCR::ABL1 p.I293_K294insSSLRD mutant detected in a Ph+ ALL patient.

Grégoire Cullot[1,2,*], Valérie Lagarde[1], Jean-Michel Cayuela[3,5], Valérie Prouzet-Mauléon[1,4], Béatrice Turcq[1,4,5], Yosr Hicheri[6], Lydia Roy[5,7] , Thorsten Braun[8], Marie-Joelle Mozziconacci[9], Anne-Sophie Alary[10#], Stéphanie Dulucq[1,5, 11,*,#]

1 - Univ. Bordeaux, INSERM, BRIC, U1312, Bordeaux, France

2 - Department of Biology, ETH Zurich, Zurich, Switzerland

3 - Laboratory of Hematology, Saint-Louis Hospital, Assistance Publique-Hopitaux de Paris, Université de Paris, University Paris Diderot, Paris, France

4 - CRISP'edit, TBMCore, CNRS UAR3427, INSERM US005, Univ. Bordeaux, Bordeaux, France

5 - Fi-LMC group, Léon Bérard center, Lyon, France

6 - Department of Hematology, Institut Paoli-Calmettes, Marseille, France

7- University Hospital Henri Mondor, AP-HP & Faculté de Santé, UPEC, Service d'Hématologie Clinique, Créteil, France

8- Department of Hematology Hospital Avicenne, Assistance Publique-Hopitaux de Paris, Bobigny, France

9 - Department of Molecular Biology, Institut Paoli-Calmettes, Marseille, France

10-. Department of Biopathology, Institut Paoli-Calmettes, Marseille, France

11-, Laboratory of Hematology, University Hospital of Bordeaux, Bordeaux, France

# - Equal contribution

** - Co-corresponding authors (gregoire.cullot@biol.ethz.ch and stephanie.dulucq@chu-bordeaux.fr)*

Figure 2b

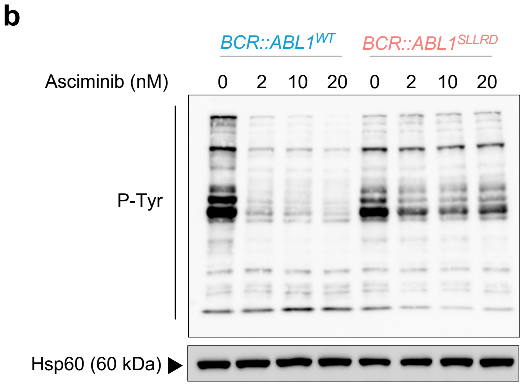
b
BCR::ABL1WT
BCR::ABL1SLLRD
Asciminib (nM)
0 2 10 20 0 2 10 20
P-Tyr
Hsp60 (60 kDa)

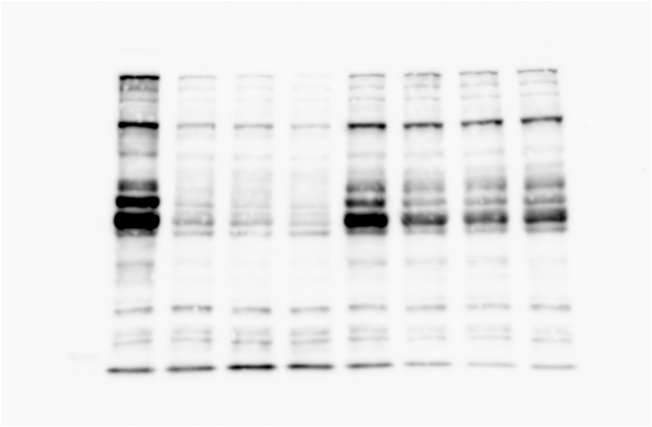

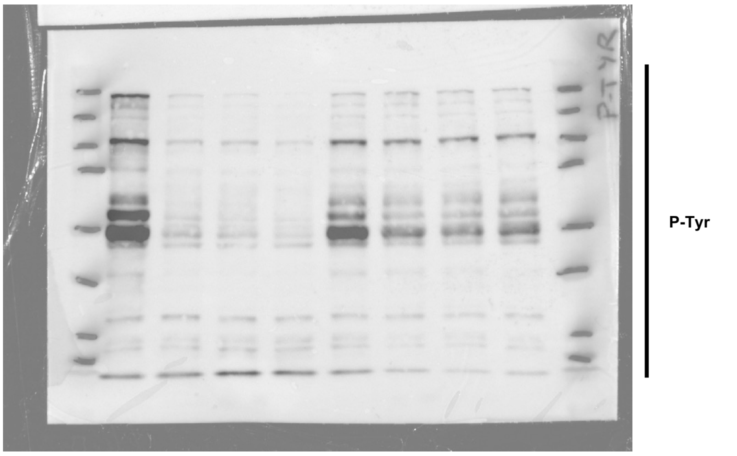
P-TYR
P-Tyr

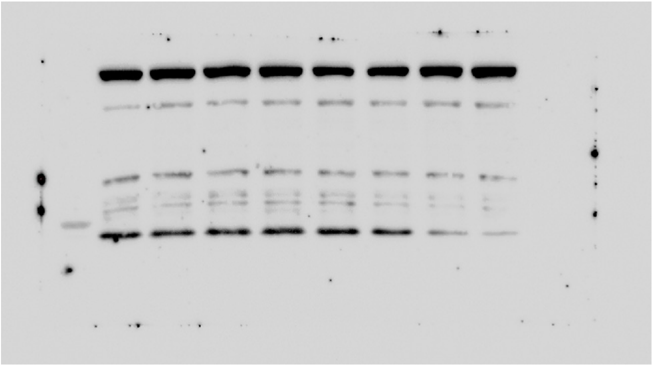

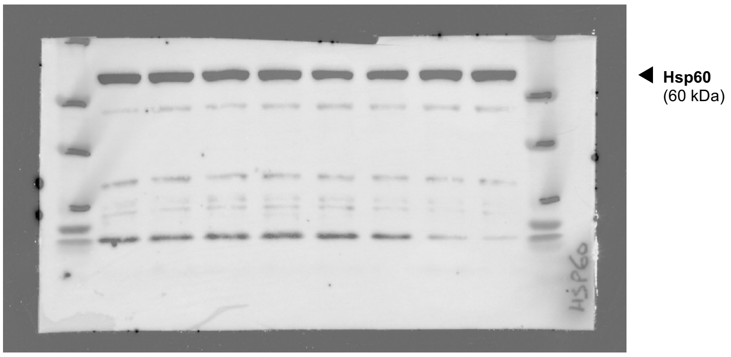
Hsp60
(60 kDa)
HSP60

Figure 2d

d

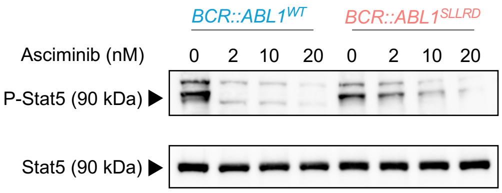

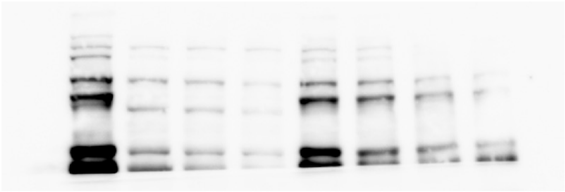

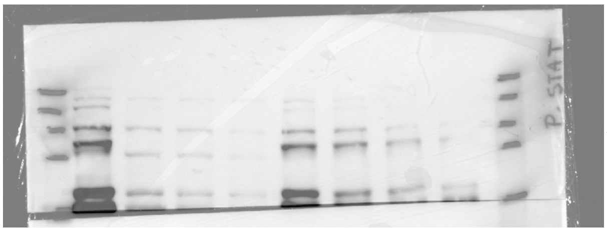

◀ **P-Stat5** (90 kDa)

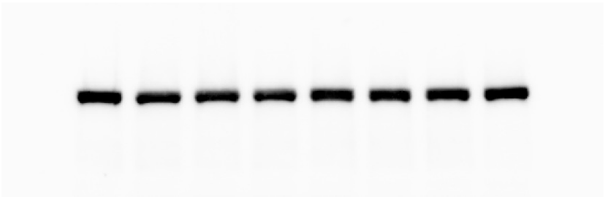

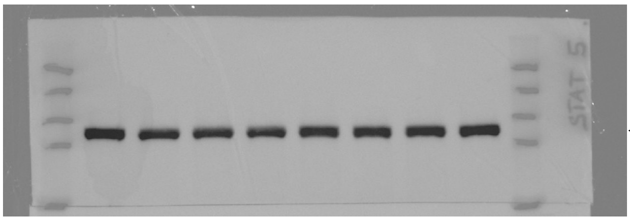

◀ **Stat5** (90 kDa)

Supplemental Figure 1c

c

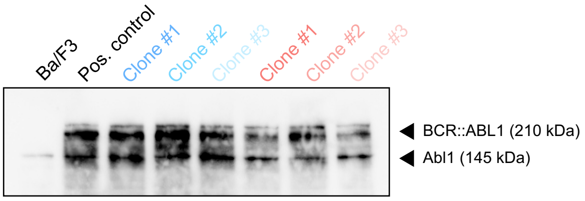

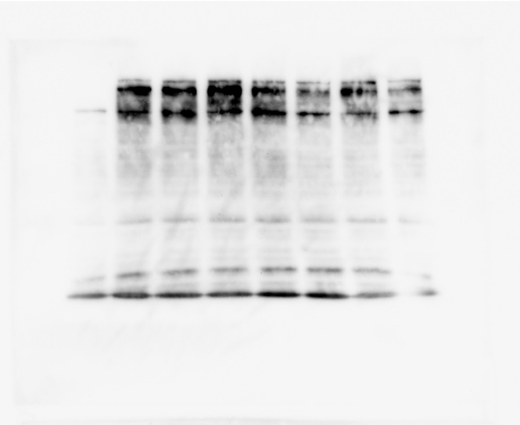

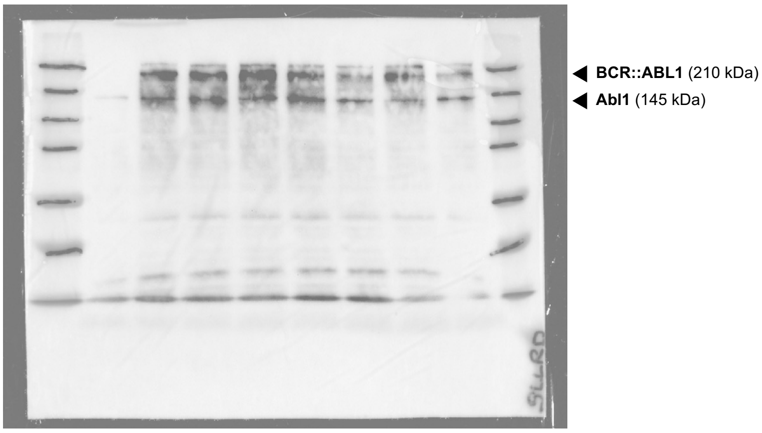

Supplemental Figure 1d

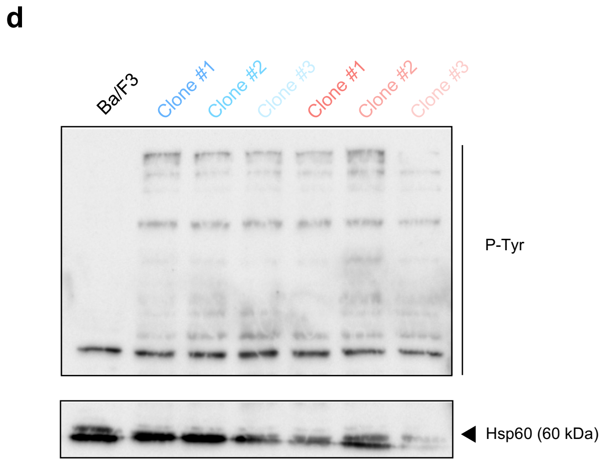

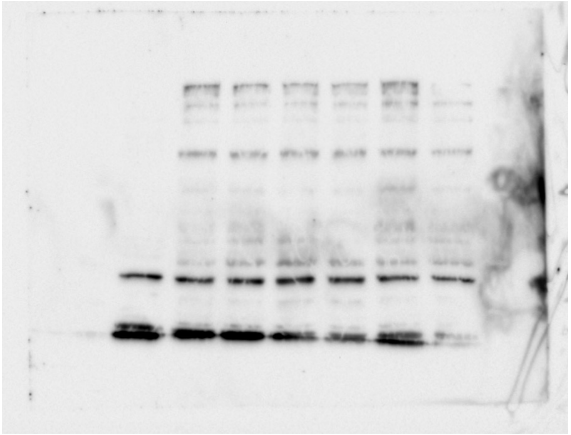

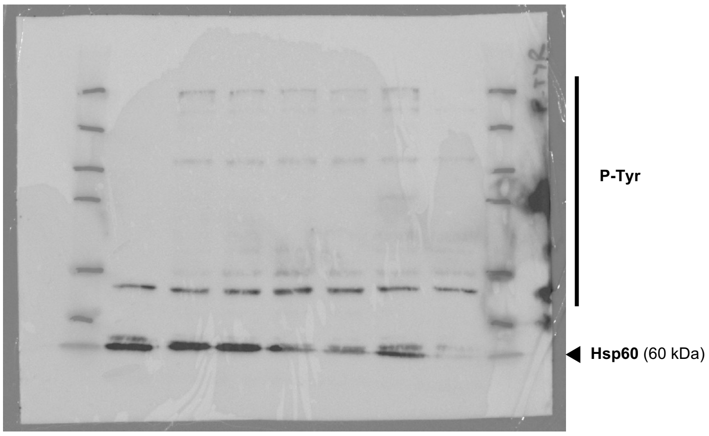

Supplemental Figure 2b

b

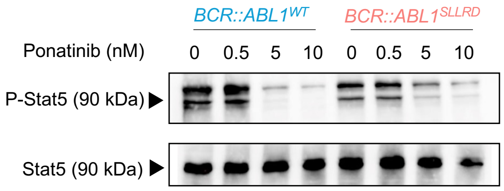

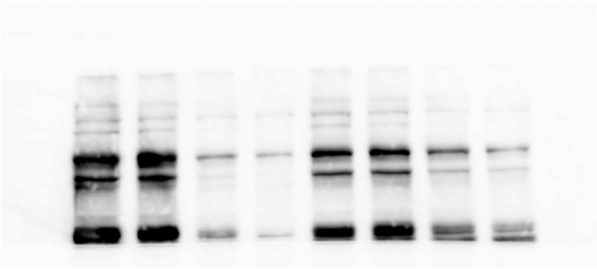

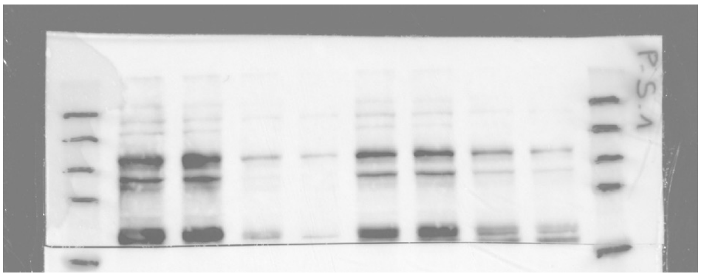

◀ **P-Stat5** (90 kDa)

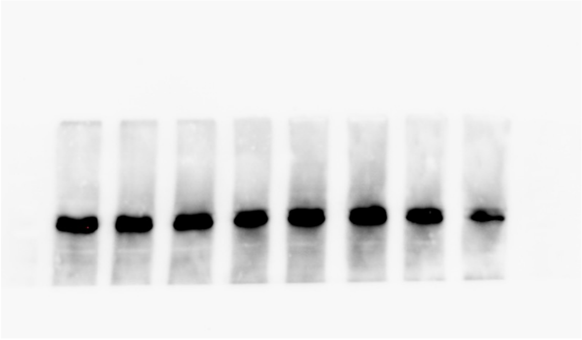

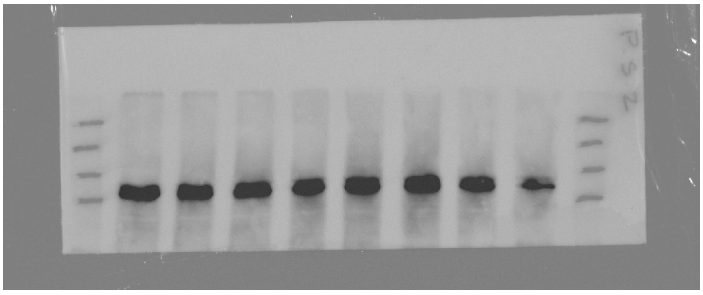

◀ **Stat5** (90 kDa)

Supplemental Figure 4b

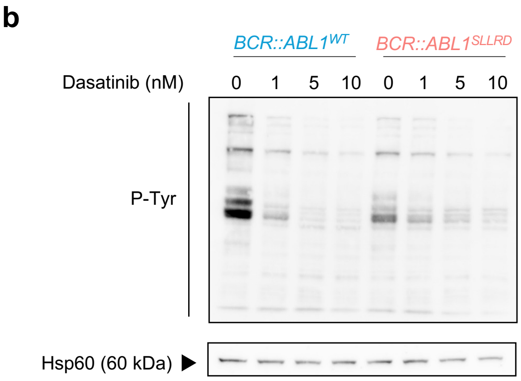

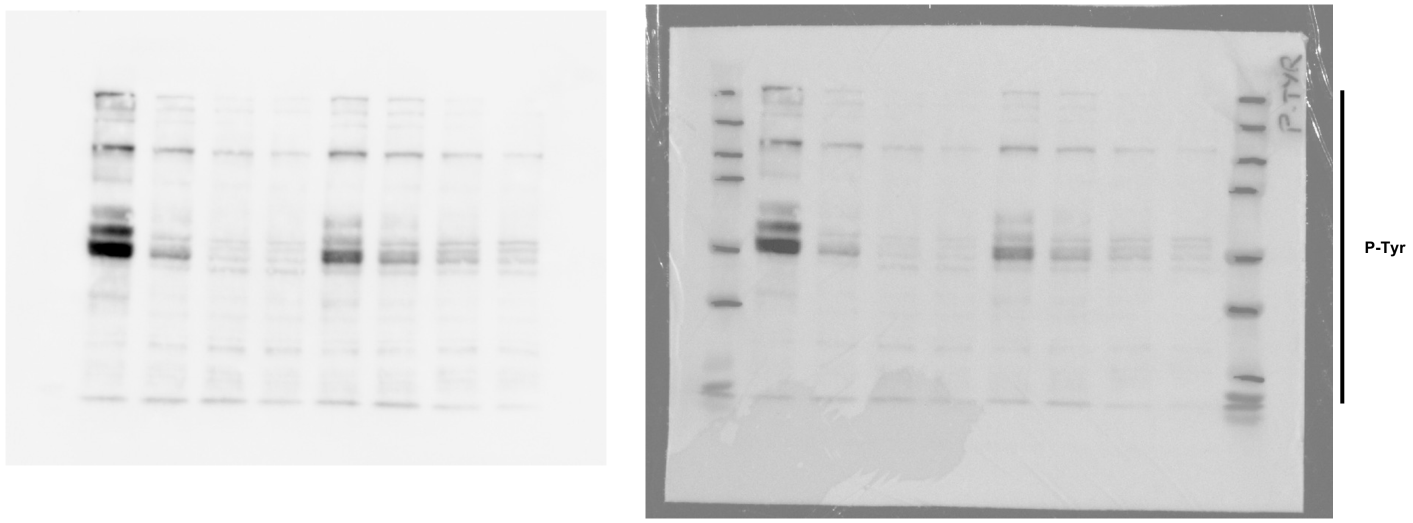

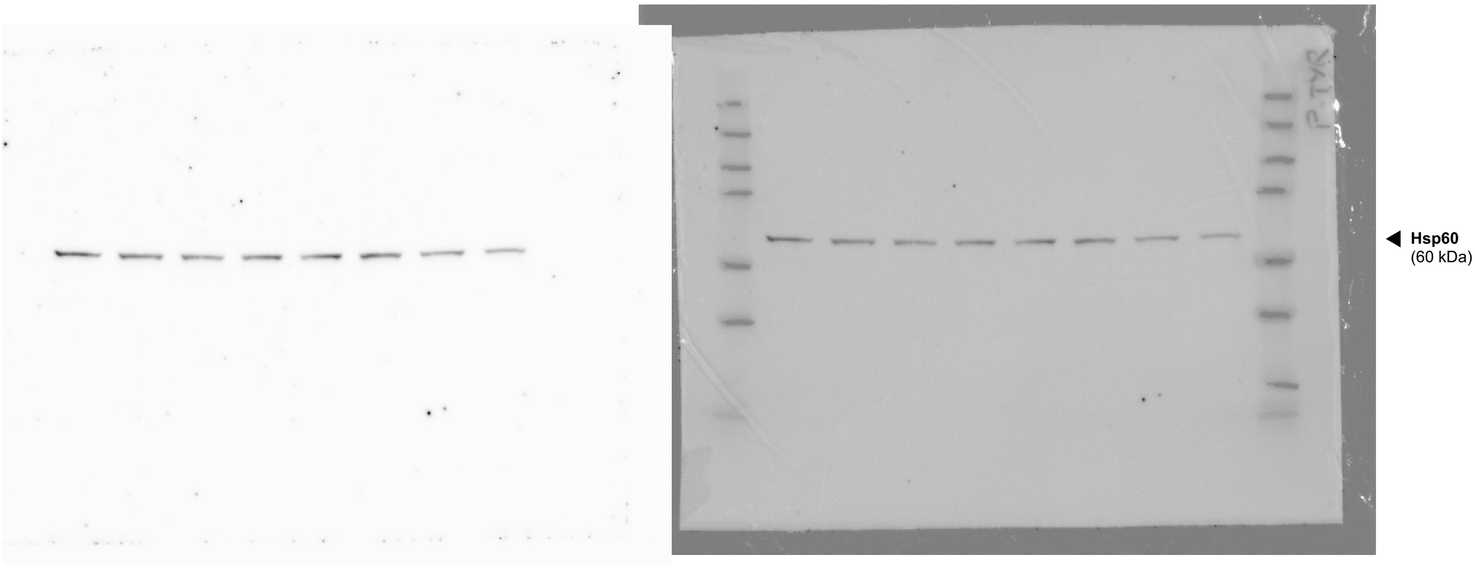

Supplemental Figure 4d

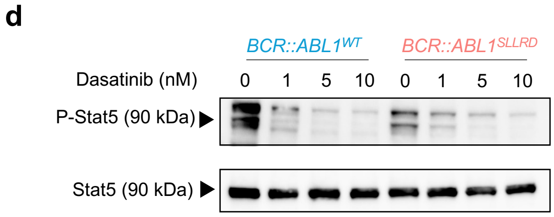

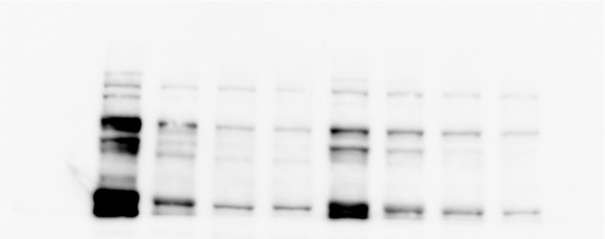

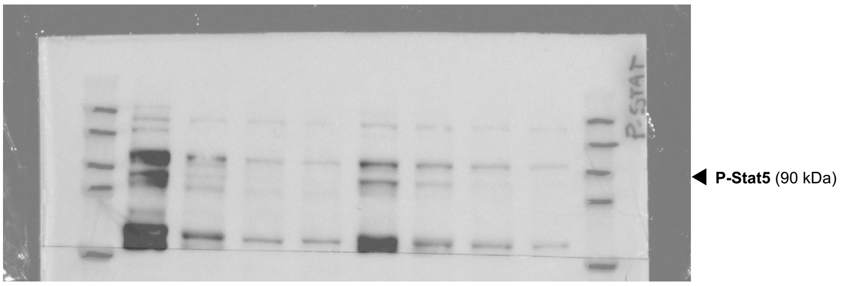

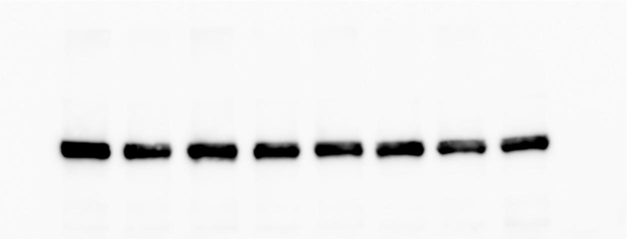

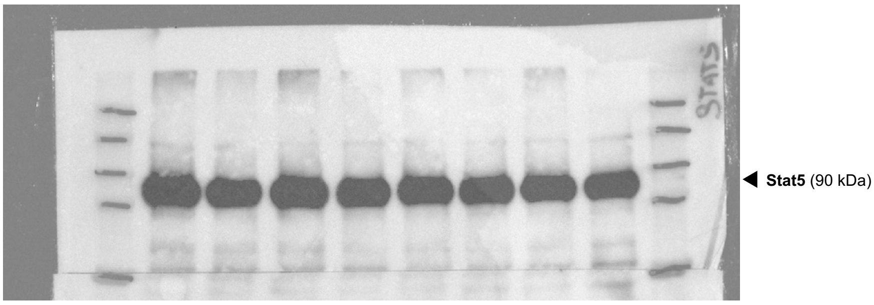

Supplement: Supplementary file 2 — Supplementary file2 (PDF 2189 KB) [file 277_2024_6142_MOESM2_ESM.pdf]
